# Females prefer the scent of outbred males: good-genes-as-heterozygosity?

**DOI:** 10.1186/1471-2148-9-104

**Published:** 2009-05-16

**Authors:** Petteri Ilmonen, Gloria Stundner, Michaela Thoß, Dustin J Penn

**Affiliations:** 1 Konrad Lorenz Institute for Ethology, Austrian Academy of Sciences, Savoyenstr. 1a, A-1160 Vienna, Austria

## Abstract

**Background:**

There is increasing interest to determine the relative importance of non-additive genetic benefits as opposed to additive ones for the evolution of mating preferences and maintenance of genetic variation in sexual ornaments. The 'good-genes-as-heterozygosity' hypothesis predicts that females should prefer to mate with more heterozygous males to gain more heterozygous (and less inbred) offspring. Heterozygosity increases males' sexual ornamentation, mating success and reproduction success, yet few experiments have tested whether females are preferentially attracted to heterozygous males, and none have tested whether females' own heterozygosity influences their preferences. Outbred females might have the luxury of being more choosey, but on the other hand, inbred females might have more to gain by mating with heterozygous males. We manipulated heterozygosity in wild-derived house mice (*Mus musculus musculus*) through inbreeding and tested whether the females are more attracted to the scent of outbred versus inbred males, and whether females' own inbreeding status affects their preferences. We also tested whether infecting *both *inbred and outbred males with *Salmonella *would magnify females' preferences for outbred males.

**Results:**

Females showed a significant preference for outbred males, and this preference was more pronounced among inbred females. We found no evidence that *Salmonella *infection increased the relative attractiveness of outbred versus inbred males; however, we found no evidence that inbreeding affected males' disease resistance in this study.

**Conclusion:**

Our findings support the idea that females are more attracted to outbred males, and they suggest that such preferences may be stronger among inbred than outbred females, which is consistent with the 'good-genes-as-heterozygosity' hypothesis. It is unclear whether this odour preference reflects females' actual mating preferences, though it suggests that future studies should consider females' as well as males' heterozygosity. Our study has implications for efforts to understand how mate choice can provide genetic benefits without eroding genetic diversity (lek paradox), and also conservation efforts to determine the fitness consequences of inbreeding and the maintenance of genetic diversity in small, inbred populations.

## Background

After considering the potential benefits of mate choice, Jerram Brown [1] decided he would "put aside the idea that there is a best male and that he is best for every female," and instead concluded that females should prefer males that genetically complement themselves, as a way to increase offspring heterozygosity or genetic diversity, which he called the "heterozygosity theory" of mate choice. Actually, Trivers [2] first suggested that females should choose mates to enhance their genetic compatibility, and this hypothesis has been supported in a variety of species [3-6]. Mating preferences for genetic compatibility, however, cannot explain why in many species females prefer males with extravagant secondary sexual traits. In another version of this model, Brown also suggested that when a "best" male is found, his superiority may be due to heterozygosity at one or more loci, and females may prefer to mate with such males to increase their offspring heterozygosity or diversity [1]. This version of the "good-genes-as-heterozygosity" hypothesis [7] has received increasing theoretical [8-13] and empirical attention [reviewed in [14]].

The main problem has been trying to explain how mating with heterozygous males could possibility provide genetic benefits. Several studies have found positive correlations between parent and offspring heterozgosity (e.g. [15-17]) and inbreeding coefficient, *f *[18], and therefore, these findings suggest that apparent non-additive genetic variation can be heritable. Such correlations arise when the frequencies of the alternative homozygous male genotypes in a population are uneven, because in such conditions homozygous females are able to increase the proportion of heterozygous offspring by mating with heterozygous males [10,12,15]. However, previous theoretical models have not explicitly addressed how females' own heterozygosity might influence their mating preferences, and have overlooked the fact that only homozygous females can increase the heterozygosity of their offspring by mating with heterozygous males (e.g., see Table 2 in [12]). Thus, females' mating preferences need not be absolute and can be conditional, depending upon their own heterozygosity. Our aims were to manipulate heterozygosity in wild house mice (*Mus musculus musculus*) through inbreeding, as this reduces genome-wide heterozygosity [19,20] by increasing the proportion of homologous alleles that are identical by descent [21,22], and test whether females are more attracted to outbred versus inbred males, and whether females' preferences depend upon their being inbred versus outbred.

Several studies have shown that heterozygosity plays a role in sexual selection [reviewed in [14]]. Male mating and reproductive success are enhanced by heterozygosity and reduced by inbreeding due to direct male-male competition [23-28]. For example, inbreeding in house mice reduces male fitness partly because it impairs males' ability to become socially dominant and maintain territories necessary to obtain mates [28-30]. Also, inbreeding may affect sperm competition as it impairs males' testicle size and sperm concentration [31,32] and decreasing heterozygosity lowers sperm quality [33], but see [34] for criticisms.

Another way that heterozygosity influences male mating success is through female preferences for heterozygous males, and a few studies support this idea [reviewed in [14]]. Maynard Smith [35], for example, found that female fruitflies (*Drosophila subobscura*) are less likely to mate with inbred than outbred males due to poor performance of inbred males during courtship. Subsequent work confirms that inbreeding or homozygosity reduces male courtship behaviour [27,36-38] and the expression of other secondary sexual traits [7,39-42], although it is unclear how inbreeding or heterozygosity affects males' attractiveness to females. Female fur seals (*Arctocephalus gazelle*) appear to seek out more heterozygous males, which they might assess through males' body size, condition, dominance behaviors, or territory quality [16] but see also [43] for criticisms. One study in Arctic charr (*Salvelinus alpinus*) suggests that heterozygous males are favored by cryptic female choice (sperm selection) [44]. Most studies have failed to find statistically significant evidence that females prefer heterozygous males [reviewed in [14]], but it is unclear whether the number of genetic markers used in these studies are sufficient to accurately assess overall heterozygosity [45]. Therefore, studies are needed that experimentally manipulate males' overall heterozygosity to test how this affects their sexual attractiveness and mating success.

Although several studies have investigated the effect of heterozygosity on male secondary sexual traits and mating success, none to our knowledge have examined whether *females' own heterozygosity *affects their preferences for heterozygous males. Several studies suggest that females' mating preferences can be condition-dependent [46-48], but only two studies, both in fish, have considered whether inbreeding affects females' mating preferences in general: the first one found that inbred females were choosier than oubred ones regarding the fluctuating asymmetry of computer-animated males [49], whereas the second found no evidence that inbreeding affects females' inbreeding avoidance [50]. Thus, inbred females may be choosier also about males' heterozygosity than outbred ones, as one would expect if they gain genetic benefits by mating with heterozygous males (e.g. see Table 2 in [12]). Inbred females may also stand more to gain in terms of *direct benefits *than outbred females by mating with high quality, heterozygous males as a way to compensate for their own poor parental quality [51-54]. On the other hand, inbred females in poor condition may not be able to afford the costs of being choosy [46-48]. Thus, studies are also needed that experimentally manipulate females' heterozygosity to test how this affects their preferences for heterozygous males.

We trapped wild house mice and inbred (sib-sib mating) the F2 generation to manipulate heterozygosity of males, and tested whether this treatment reduces their attractiveness to females in comparison to outbred males in an olfactory preference assay. Females were presented with males' scent-marks, which are a testosterone-mediated, condition-dependent secondary sexual trait used in courtship [55]. We also manipulated the heterozygosity of females through inbreeding to test whether this affects their preferences for outbred males. Often, the detrimental effects of inbreeding only become apparent after exposure to infectious agents, social competition, or other stressful conditions [27,28,30,56]. Therefore, in each trial, we tested females' preferences for inbred versus outbred males when *both males *had been experimentally infected with *Salmonella *or *both *were sham-infected. The infection treatment was performed to make a negative result more conclusive, and if inbreeding reduces males' attractiveness due to their relatively poor health and condition, then we predicted that infection would magnify the differences between males. In fact, *Salmonella *infection has been found to magnify the fitness differences between inbred versus outbred males [30]. We found that females show a strong and clear preference for the scent of outbred males, regardless of whether the both of the males were experimentally infected or not, and this preference was somewhat stronger among the inbred females.

## Methods

### Animals and housing

We trapped wild house mice from a single population (Safaripark, Gänserndorf) near Vienna, Austria and bred the F2 generation to produce full-sib inbred (sister-brother-mating; Wright's inbreeding coefficient; *f *= 0.25) and outbred mice (matings between unrelated individuals; *f *= 0.00). At weaning, we housed the offspring singly in acrylic cages, half of the inbred and outbred males in type I cages (22 × 16 × 14 cm) and the other half in type IIL cages (32.5 × 16 × 14 cm, IVC). The females were housed in type IIL cages. The cages contained pine bedding and wood-wool for environmental enrichment. All the mice were provided food (Altromin rodent diet 1324) and water *ad libitum *and kept under a 12:12 h dark:light cycle. For the odour preference test, we chose 52 triplets (one inbred male, one outbred male and one female) in which the three mice were closely age-matched, unrelated and unfamiliar to each other. All mice were sexually mature. Experimental protocol was approved by the Austrian Federal Ministry of Science and Research' Animal Care and Use Committee (BMWF-66.015/0023-c/GT/2007).

### Experimental infections

The 52 males (26 inbred and 26 outbred males) of the infection group were experimentally infected with 30 μl of *Salmonella enterica *serovar Typhimurium [strain SRI – 11, 10^6 ^colony forming units (cfu)/ml] orally, which is a natural infection route. *S. enterica *serovar Typhimurium is an enteric mouse pathogen that becomes systemic by invading the intestinal mucosa and by replicating intracellularly within host macrophages [57]. Host resistance to *Salmonella *is under genetic control and influenced by nramp, major histocompatibility complex and other immune resistance loci [58], and requires both innate and acquired arms of the immune system [59]. We used *Salmonella *as an experimental pathogen as our previous work found that inbreeding increases the susceptibility of mice to *Salmonella *[30], and *Salmonella *infection reduces male scent-marking and the attractiveness of males' scent to females [55]. Therefore, we predicted that female preferences for outbred versus inbred males would be more pronounced when *both *of the males were experimentally infected with *Salmonella *(and if the results were negative, this treatment would allow us to conclude that this result was not an artefact of the males not being infected or otherwise challenged, as occurs in more normal ecological circumstances). The bacteria (stored as frozen stocks at -80°C) were cultured in 15 ml of heart-brain infusion at 37°C for 12 h while shaking at 170 rpm. The overnight solution was diluted to the desired concentration with sterile phosphate buffered saline (PBS) and the concentration of viable bacteria was verified by quantitative plate counts in duplicates. After infection, the mice were housed singly and euthanized 11-days post inoculation with CO_2_. The mice were inspected on a daily basis and the individuals that showed clear symptoms of severe infection were euthanized immediately to avoid any unnecessary suffering. The spleens of the mice were dissected and homogenized in 1 ml of PBS under sterile conditions. 50 μl of each homogenate was cultured on selective agar plates and incubated overnight (37°C). The *Salmonella *loads per spleen were determined by calculating the number of cfu/ml of spleen homogenates on the plates (the mean of two replicate plates per mouse). The mice were restricted from food and water four hours prior to inoculation to rule out variation in systemic infection due to food in the gut. Three of the *Salmonella*-infected males died before the scent mark collection and therefore we could not perform any odour preference tests with these triplets. The 52 males (26 inbred and 26 outbred males) of the control group were sham-infected by given them equal volume of sterile PBS. We used a lower *Salmonella *dosage here than in a previous study, and therefore, we expected lower mortality, especially since in a previous study most mortality occurred only after the mice had been repeatedly challenged with mixed strain infections over several months [60]; however, mortality was unexpectedly 10% higher in this study.

### Scent-mark collection

To collect the scent marks, we placed the males into a new small cage on a sterile filter paper (20.5 × 14.5 cm) for four hours eight days after inoculation. We collected scent marks in the morning (8:00–12:00 a.m.). During this time, males were provided food and water *ad libitum*. Males were stimulated with female urine because stimulated males show more scent-marking and females show a preference for scents of sexually stimulated males [55]. We placed a small piece of filter paper (2 × 2 cm) containing 10 μl of female urine into the males' cages. We used mixture of urine from 15 mature females (different from those used in the odour preference tests), which we collected by placing females on tinfoil, pipetting up the urine, and storing it at -80°C. The filter papers with male scent marks were stored individually in Ziploc^® ^plastic bags (Toppits, Allround Zipper, 3 l) at -80°C until used in female odour preference tests. The cages of the males were filled with new bedding after the scent mark collection so that they felt comfortable in their cages (normal weekly animal care taking which we connected with the experiments). This way, 46 marked filter papers of infected males (23 inbred, 23 outbred) and 52 marked filter papers of sham-infected males (26 inbred, 26 outbred) could be generated.

### Odour preference assays

We tested females during oestrus, determined by examining vaginal smears under a microscope [61], to ensure they were sexually active. The Y-maze apparatus for our odour preference tests was composed of acrylic, and contained a start chamber (5.5 × 12.5 × 5.5 cm), where the mice were first placed, and two arms of choice chambers. The start chamber was separated from the first section of the choice chambers or neutral zone, and the choice chambers (5.5 × 13.5 × 5.5 cm; without neutral zone) were separated from the chambers containing the filter papers (5.5 × 31.5 × 5.5 cm) with wire-mesh dividers. The dividers prevented the females from touching or chewing the filter papers. We placed an air pump (Sera Air 110) and the scent marked filter papers at the end of the chambers to ensure a constant airflow of volatiles through the maze. The pump was kept constantly on in the colony room to habituate the females to its sound.

The experiments were conducted in the morning beginning at 8:00 a.m. under dim light, recorded on videotape (Sony Handycam DCR-SR 30E) and the videos were later analysed using Observer software (Noldus, Version 3). At the start of each trial, a female was placed in the start chamber for 5 min to habituate to the maze, and after this time the scent-marked filter papers were placed in the maze. The air pump was turned on and the female was released into neutral zone of the choice chambers. Based on preliminary tests, we recorded the females for 5 min because thereafter they were less active. We recorded the following behaviours: (1) the number; and (2) the duration female actively investigated the dividers between the choice chambers and the chambers containing the filter papers; and (3) the number of visits; and (4) total time a female spent on each side of the Y-maze. We predicted *a priori *that the two investigatory behaviours (1 and 2) would be the most informative for female preferences, because the females actively gather information and show interest in the odour. The other two behaviours (3 and 4) were recorded because these are commonly used in preference tests. We considered any side biases females showed to indicate an odour preference. After each trial, the Y-maze was cleaned with ethanol (to remove scents from previous trial), and we alternated the sides of the maze in which the filter papers were placed (between inbred versus outbred males, infected versus sham-infected pairs of males, and inbred versus outbred females) to avoid biases due to possible side-preferences. Each filter paper and each female was tested only once. To avoid possible experimenter biases, there was only one observer who recorded the data (videotape playbacks) and she was blind to the inbreeding and infection status of the animals.

### Statistical analyses

We tested the data for assumptions of normality and equality of variances before conducting parametric tests (SPSS version 15.0). For statistical analyses, we used General Linear Model (GLM), repeated measures. The tests of within-subjects effects was used to test whether there was a general preference for outbred versus inbred males, and whether the female inbreeding status or male infection status (both *Salmonella*-infected or sham-infected males) had any influence on female preference. The between-subjects effect was used to test whether the female inbreeding status or male infection status influenced female behaviours. We ran paired samples *t*-test separately for inbred and outbred females, but only for the number of investigations, because the interaction term with female inbreeding status was statistically significant only for this variable. Furthermore, all of the four female behaviours were highly inter-correlated (*R *> 0.47, *N *= 49, *P *< 0.001, for all pair-wise correlations). We used directed tests instead of one- or two-tailed tests [62] for the overall female preference for outbred males over inbred males, because we had a clear *a priori*-prediction that females would prefer the outbred males over the inbred ones, which is consistent with previous results [14]. We also used directed test for testing the effect of male infection status on female preference because we predicted *a priori *that female preference for outbred male is more pronounced when both males are experimentally infected. To test for differences in the *Salmonella *loads we used a *t-*test (log_10_-transformed data) and to test for differences in the prevalence (infected or non-infected) and the mortality between inbred and outbred males we used Chi-square tests. We used directed tests because in a previous study it was found that the outbred males are more resistant to *Salmonella *than the inbred ones [30]. We obtained the critical values for each directed test from the *P*-values of the corresponding one-tailed test by using γ/α = 0.8 as a pragmatic conventional value [62]. Using two-tailed tests instead of directed tests does not change the interpretation of our results, except that the observed female preference for outbred versus inbred males measured by duration of investigations becomes only marginally significant (*P *= 0.05).

## Results

**Figure 1:**
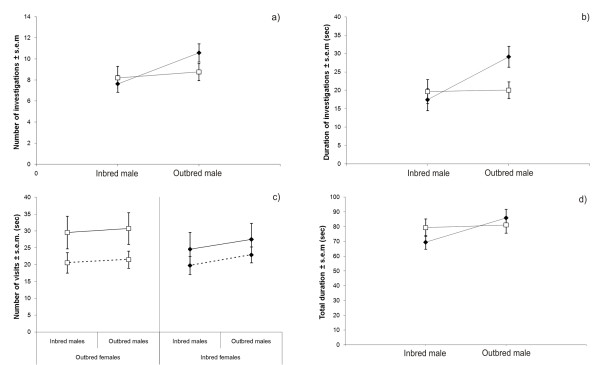
**Female preferences measured as a) number of investigations, b) duration of investigations, c) number of visits and d) total duration separately for outbred (white symbol, n = 25) and inbred females (black symbol, n = 24)**. Data is pooled for trials in which both of the males were sham-infected or both infected, except for 1 c, in which the data is shown separately for trials with two sham-infected males (dashed line) and two infected males (solid line).

The results of GLM multivariate analysis showed that females preferred significantly outbred males over the inbred ones [Within-subjects effects, outbred (OB) versus inbred (IB) male: F = 3.0, d.f. = 4, *P*_*dir *_= 0.02] measured by average of the four female preference behaviours, whereas neither the female inbreeding status (interaction term: OB versus IB male × female inbreeding status: F = 1.6, d.f. = 4, *P *= 0.19) or experimental infection (interaction term: OB vs IB × male infection status: F = 0.2, d.f. = 4, *P*_*dir *_= 0.59) had no significant effects on female preference for outbred males. When using univariate models we found that females significantly preferred outbred compared to inbred males, measured by number of investigations (Table 1, Fig. 1a), duration of investigations (Table 2, Fig. 1b) and number of visits (Table 3, Fig. 1c), but not by total duration (Table 4, Fig. 1d). Interestingly, we found that preference for outbred males was somewhat stronger in inbred females versus outbred females (Figs 1a–d). This difference between inbred and outbred females was statistically significant for number of investigations (Table 1; Within-subjects contrasts, interaction term: OB versus IB male × female inbreeding status), and there was a similar, but non-significant trend for duration of investigations. Females' inbreeding status did not influence their preferences for number of visits (Table 3) or total duration (Table 4). When testing the inbred and outbred females separately, inbred females investigated the scent marks of outbred males significantly more often compared to the scent marks of inbred males (paired samples *t-*test, *t *= 4.50, d.f. = 23, *P*_*dir *_= 0.0001), but outbred females did not show any clear preference (paired samples *t-*test, *t *= 0.79, d.f. = 24, *P*_*dir *_= 0.44, Fig. 1a).

**Table 1:** Summary table for the results of GLM repeated measurements analyses for number of investigations.

**Tests of Within-Subjects Contrasts**	**d.f.**	***F***	***P***
preference OB vs IB (directed)	1	12.5	**0.0006**
preference OB vs IB × female inbreeding status	1	6.1	**0.017**
preference OB vs IB × male infection status (directed)	1	0.0	0.56
preference OB vs IB × female inbreeding status × male infection status	1	0.7	0.42
Error (OB vs IB)	45		

**Tests of Between-Subjects Effects**	**d.f.**	***F***	***P***

female inbreeding status	1	0.3	0.57
male infection status	1	0.1	0.78
female inbreeding status × male infection status	1	1.0	0.32
Error	45		

(OB = outbred; IB = inbred).

**Table 2:** Summary table for the results of GLM repeated measurements analyses for duration of investigations.

**Tests of Within-Subjects Contrasts**	**d.f.**	***F***	***P***
preference OB vs IB (directed)	1	4.0	**0.033**
preference OB vs IB × female inbreeding status	1	3.3	0.08
preference OB vs IB × male infection status (directed)	1	0.0	0.53
preference OB vs IB × female inbreeding status × male infection status	1	0.1	0.75
Error (OB vs IB)	45		

**Tests of Between-Subjects Effects**	**d.f.**	***F***	***P***

female inbreeding status	1	1.7	0.20
male infection status	1	0.8	0.39
female inbreeding status × male infection status	1	0.2	0.63
Error	45		

(OB = outbred; IB = inbred).

**Table 3:** Summary table for the results of GLM repeated measurements analyses for number of visits.

**Tests of Within-Subjects Contrasts**	**d.f.**	***F***	***P***
preference OB vs IB (directed)	1	8.0	**0.004**
preference OB vs IB × female inbreeding status	1	1.9	0.18
preference OB vs IB × male infection status (directed)	1	0.0	0.62
preference OB vs IB × female inbreeding status × male infection status	1	0.0	0.86
Error (OB vs IB)	45		

**Tests of Between-Subjects Effects**	**d.f.**	***F***	***P***

female inbreeding status	1	0.3	0.62
male infection status	1	3.4	0.07
female inbreeding status × male infection status	1	0.3	0.56
Error	45		

(OB = outbred; IB = inbred).

**Table 4:** Summary table for the results of GLM repeated measurements analyses for total duration

**Tests of Within-Subjects Contrasts**	**d.f.**	***F***	***P***
preference OB vs IB (directed)	1	2.3	0.08
preference OB vs IB × female inbreeding status	1	1.3	0.27
preference OB vs IB × male infection status (directed)	1	0.4	0.32
preference OB vs IB × female inbreeding status × male infection status	1	0.1	0.80
Error (OB vs IB)	45		

**Tests of Between-Subjects Effects**	**d.f.**	***F***	***P***

female inbreeding status	1	0.1	0.72
male infection status	1	1.8	0.19
female inbreeding status × male infection status	1	2.3	0.14
Error	45		

(OB = outbred; IB = inbred).

We found no significant evidence that infecting both the inbred and outbred males influenced their relative attractiveness: the females still preferred outbred males, but contrary to our expectation this preference was not magnified when both of the males were experimentally infected (Tables 1, 2, 3, 4). Interestingly, the females showed a tendency to shift the sides within the Y-maze more often (number of visits, Fig. 1c) when they were presented with the scents from two infected compared to two sham-infected males; however, this difference is not significant (the between-subjects effects: male infection status; Table 3). One possible reason we did not find that infection would magnify females' preferences for outbred males is that inbreeding did not appear to affect the males' resistance to *Salmonella *infection in this experiment. There was a statistically non-significant trend for lower mortality in the outbred males compared to the inbred ones (27% and 46%, respectively; Chi-square test, χ^2 ^= 2.07, d.f. = 1, *P*_*dir *_= 0.09). However, among the survivors, there were no statistically significant differences in *Salmonella *loads between inbred and outbred males after eleven days (log_10 _*Salmonella *load: 3.00 ± 0.83 and 2.64 ± 0.72, respectively; Independent samples *t-*test, *t *= 0.33, d.f. = 31, *P*_*dir *_= 0.47). Although many mice completely cleared the infection, there was no difference in *Salmonella *prevalence (57% and 47%, respectively; Chi-square test, χ^2 ^= 0.31, d.f. = 1, *P*_*dir *_= 0.36). Thus, our experimental infection did not increase the females' preferences for outbred males.

## Discussion

We found that female mice were more attracted to the scent marks of outbred compared to inbred males, as predicted, and this preference appeared to be more pronounced among the inbred females compared to outbred ones. Our findings suggest that female house mice may prefer to mate with heterozygous males, and especially so if they have reduced heterozygosity themselves, which suggests a novel version of the 'heterozygosity-as-good-genes' hypothesis. Since we controlled for male-male interactions, our results cannot be due to outbred males being more socially dominant, and females simply preferring dominant males [63]. We suspect that inbreeding reduced the health and condition of the males, but we found no evidence that an experimental *Salmonella *infection increased the relative attractiveness of the outbred males. However, we cannot rule out the possibility that infection or other stressors would magnify the differences because, surprisingly, inbreeding had no detectable effect on the males' pathogen clearance in this study. When the two males in a trial were both infected (experimental infection-group), we found that the females tended to move between the males more frequently than when the males were both uninfected (sham-controls). This difference was not statistically significant (*P *= 0.07), but it suggests that the females may have more difficulty distinguishing the quality of the males when they are both infected, which is the opposite of what we assumed.

Our findings raise the possibility that inbred, homozygous females may gain more genetic (fitness) benefits by mating with heterozygous males compared to outbred, heterozygous females (see Table 2 in [12]). Most previous theoretical models do not support the idea that mating with heterozygous males will increase female fitness, or contribute to maintaining genetic variation in male traits or female preferences (the so-called 'lek paradox') (reviewed in [10]). Some have suggested that the model might work in fluctuating environments [1,8,9], or in small populations with genetic drift [10,11]. These conditions might be more realistic than often assumed, and especially so for species like house mice that live in small demes consisting of related individuals [64]. Two recent papers that incorporated finite population size and genetic drift [12] or populations with spatial genetic structure [13] found that inbreeding co-efficient (*f*) or heterozygosity can be inherited, that female mate choice for outbred or heterozygous males can evolve, and that the "heritability" of *f *or heterozygosity, and hence the non-additive benefits for females, are highest in small populations. However, like [10], these models assume that heterozygotes have higher fitness due to overdominance, which is extremely rare (and observations of heterozygote advantage can be due to dominance rather than overdominance, and experiments support this interpretation [65]), and therefore, unlikely to provide a general solution. Our findings suggest that future models should incorporate the possibility that female preferences may be conditional depending upon their own heterozygosity. In genetically structured populations, heterozygous males may be more likely to carry locally rare and dissimilar alleles, which could be particularly important for homozygous females to increase offspring heterozygosity and reduce inbreeding (see also [66] and [18]). Females may gain other types of genetic benefits by mating with heterozygous males, such as increasing the within-brood genetic diversity of offspring [1,67,68], or optimizing the heterozygosity of offspring [69-71].

On the other hand, mating with heterozygous males may provide no genetic benefits for females; however, as previously mentioned, it may provide *direct benefits*. For example, in house mice, outbred males defend territories more effectively than inbred ones [28], which should reduce the risks of infanticide and sexual harassment by other males, and in other species, improve parental care. Such direct benefits might be relatively more important to inbred females since they are poorer parents than outbred females [51-54].

Our findings also raise questions about the proximate mechanisms controlling males' scent-marking behaviour and females' odour preferences. They indicate that inbreeding alters males' scent-marks, either by reducing the quantity or quality of marks they produce. Condition-dependent sexually selected traits are thought to be especially vulnerable to negative inbreeding effects, because male's overall condition and health is influenced by multiple genes, and hence provide a large mutational target [72], which is why outbred, heterozygous, males are expected to be able to invest more into costly secondary sexual traits [73]. We suspect that inbred males have lower androgens than outbred males, and subsequently reduced scent-marking, androgen-dependent Major Urinary Proteins (MUPs) and sexual pheromones in their urine. This seems likely since inbreeding impairs males' testicle size and function [31-33], courtship behaviour [27,36-38] and the expression of other secondary sexual traits [7,39-42]. It is difficult to understand why low quality males do not 'cheat' and produce more attractive scent-marks, unless scent-marking is costly and low quality males cannot afford the costs (handicap or costly signalling hypothesis), or unless it is physiologically impossible for males to produce compounds in their urine that would disguise poor health or condition [55,74-76]. The idea that quality of males' scent marks is influenced by heterozygosity, and thus potentially allow females to distinguish males with different levels of genetic diversity and relatedness, is supported by a recent study in ring-tailed lemurs (*Lemur catta*) that found that the chemical composition of males' odour reflects marker-based heterozygosity [77]. Moreover, inbred females may be more likely to recognize heterozygous males because, as we previously pointed out, such males may carry dissimilar and unfamiliar (locally rare) alleles, at least in genetically structured populations, such as found in house mice [64]. If female odour preferences are based on phenotypic matching, it should be an easier olfactory task for homozygous females to recognize novel and dissimilar alleles carried by heterozygous males than for heterozygous females. A recent study found that wild-derived females prefer to associate with male mice derived from crosses of laboratory strains that were heterozygous at markers linked to MUP genes [78], but it is unclear whether this is due to differences in males' *scent*. Also, the males in this study were allowed to interact before the trials, which might explain the results, as females prefer the scent of dominant males [63]. We would expect that male-male interactions would magnify differences in the attractiveness of homozygous versus heterozygous males [28], but this idea has not been tested. It would be interesting to know if females' preferences are influenced by their own MUP heterozygosity, and whether such preferences are affected by their own condition.

## Conclusion

To conclude, our findings provide experimental support for the 'good-genes-as-heterozygosity'-hypothesis by showing that female mice prefer outbred males over the inbred ones. Furthermore, our results imply that this preference could be stronger among inbred females, which is in good agreement with predictions that inbred females have more to gain by preferring heterozygous males. Thus, there appears to be no 'best' strategy for every female when choosing among males with different heterozygosity levels. It is unclear from our study whether the females' preferences for scent-marks predict their actual mating preferences in the wild, but if so, our results have important implications for several issues in behavioural ecology, evolutionary biology, and conservation biology. Firstly, our findings suggest that mating preferences could help explain why inbred males have such a low reproductive success when they must compete for mates [27,28,30]. Secondly, our results contribute to the current debate on sexual selection theory and, in particular, how the non-additive genetic benefits could maintain additive genetic variance in male secondary sexual traits and consequently directional mating preferences in females, which has been advocated at least as a partial resolution to the lek-paradox ([13,16,12,66], but see [43,10] and [11] for criticisms). Lastly, our results suggest that female preferences for heterozygous males may provide a selective factor against inbred males expressing deleterious, recessive mutations, and thus could help to maintain genetic diversity in endangered small populations (see also [17,10,12,79]).

## Authors' contributions

PI, GS and DJP conceived and designed the experiments. GS, MT and PI carried out the experiments, and PI ran the statistical analyses. PI and DJP wrote the paper. All authors read and approved the final manuscript.
